# The effect of psilocybin on empathy and prosocial behavior: a proposed mechanism for enduring antidepressant effects

**DOI:** 10.1038/s44184-023-00053-8

**Published:** 2024-02-20

**Authors:** Kush V. Bhatt, Cory R. Weissman

**Affiliations:** Department of Psychiatry, University of California, San Diego, La Jolla, CA USA

**Keywords:** Human behaviour, Depression, Empathy, Social behaviour

## Abstract

Psilocybin is a serotonergic psychedelic shown to have enduring antidepressant effects. Currently, the mechanism for its enduring effects is not well understood. Empathy and prosocial behavior may be important for understanding the therapeutic benefit of psilocybin. In this article we review the effect of psilocybin on empathy and prosocial behavior. Moreover, we propose that psilocybin may induce a positive feedback loop involving empathy and prosocial behavior which helps explain the observed, enduring antidepressant effects.

## Introduction

Psilocybin is a serotonergic psychedelic currently being considered for the treatment of several psychiatric illnesses including depression^1–6^. As psilocybin research rapidly expands, important questions about the mechanism by which psilocybin exerts its antidepressant effects remain unanswered. A question of particular interest is how psilocybin achieves enduring antidepressant effects well beyond the expected physiological metabolism of the drug. While the half-life of psilocybin is ~3 h^7^, antidepressant effects of a single dose have been shown to last for up to 6 months^8,9^. Animal and human studies of psilocybin have attempted to shed light on a mechanism by which psilocybin may be causing a therapeutic benefit. Animal models have shown that classic serotonergic psychedelics, including psilocybin, require 5HT2A receptor activity for psychoactive effects^10^. Further, preclinical studies have suggested that psilocybin may induce BDNF protein synthesis and subsequent downstream neuroplastic changes in a TRK-B receptor-dependent manner^11^

Human studies have confirmed the role of 5HT2A receptor agonism for the psychoactive effects of psilocybin^12^ and suggest that psilocybin may induce modulation of brain networks. For example, modulation of the default mode network has been associated with the therapeutic effects of psilocybin in human subjects^13,14^.

While research continues to investigate physiological mechanisms through which psilocybin induces therapeutic effects, many experts propose that the subjective psychedelic experience, often termed the mystical experience, is a necessary component^15^. Studies have demonstrated correlation between acute subjective mystical experiences and enduring positive psychological changes. For example, one study of healthy participants found that when high-dose psilocybin induced a mystical experience, it produced positive changes in interpersonal relations, gratitude, and life purpose at 6-month follow-up^16^. Similar findings were shown in another study that found psilocybin occasioned mystical experience produced positive changes in mood, attitude, behavior, and social effects at 3 months^17^. Another study found that psilocybin-induced mystical experience was associated with increases in trait mindfulness at 3-month follow-up^18^. Psilocybin-induced mystical experience has also been associated with reductions in depressive symptoms, as measured by the Quick Inventory of Depressive Symptoms Self Report (QIDS-SR) at 5 weeks after psilocybin treatment^19^.

A greater sense of connectedness and unity with others is a key quality of the mystical experience and is thought to be involved in the antidepressant effect of psilocybin^20^. Greater sense of connected has also been linked with empathy and prosocial behavior^21–24^. Psilocybin has been shown to induce significant alterations in social processing; moreover, the effect of psilocybin on social functioning at an individual and community level may be vital for a comprehensive understanding of its mechanism of action and therapeutic potential^25–27^. Thus, it is worth considering empathy and prosocial behavior as processes implicated in the therapeutic effects of psilocybin. In this article, we review the literature on the effects of psilocybin on empathy and prosocial behavior. Moreover, we suggest that psilocybin may catalyze a positive feedback loop involving empathy and prosocial behavior which, in part, may lead to enduring therapeutic benefit.

## Exploring the relationship between empathy and prosocial behavior

There is no consensus definition of empathy; however, it can be broadly defined as the ability to adopt the perspective of others^28,29^. Empathy can be categorized as either cognitive empathy or emotional empathy^30^. Cognitive empathy is the mental process of understanding another individual’s perspective. It does not necessitate an affective response and is an analytical process. In contrast, emotional empathy is defined as one’s ability to recognize and feel the emotions of another. In other words, it is an induction of an affective state in response to the experience of others^30^. Emotional empathy can be further divided into positive and negative emotional empathy, defined as empathizing with the positive experiences in others versus empathizing with the distress of others, respectively^31^.

Research has explored neural correlates of empathy both in humans and animal models and various brain regions have been implicated. While not a comprehensive list, this includes the prefrontal cortex, amygdala, nucleus accumbens, anterior cingulate, and insula^32,33^. One interesting finding is that differential activity appears to be dependent on context and affective valence (i.e., positive empathy vs. negative empathy). For example, positive empathy may be associated with activity in the medial orbitofrontal cortex, while negative empathy with activity in the right insulo-frontal cortex^34^. Furthermore, it appears there may be some overlap between empathy and psilocybin in regard to neural correlates. A study using a mouse model of fronto-temporal dementia demonstrated that loss of empathy was associated with hypo-excitability of the dorsal medial prefrontal cortex^35^. Empathy training in adolescents has been shown to cause increased activity in the temporoparietal junction. Interestingly, psilocybin has been associated with activity and increased neuroplasticity of the prefrontal cortex^36,37^, as well as in the temporal parietal junction^38^.

Prosocial behavior, defined as behavior by an individual that attempts to increase the welfare of another, is known to be catalyzed by empathy^39^. It has been thought that there is a strong, positive relationship between empathy and prosocial behavior^29,40^. However, some studies have brought this into question, such as when empathy induces overwhelming personal distress or prosocial efforts feel personally onerous, in which case empathy may not produce prosocial behavior^28^. These mixed findings suggest a more complex relationship between empathy and prosocial behavior which may be dependent on various mediating factors. In discussing the mechanism of action of psilocybin, it is worth exploring the role of positive affect in the relationship between empathy and prosocial behavior, as psilocybin has been shown to produce enduring positive affect^16^.

Some research suggests that positive affect can facilitate the relationship between empathy and prosocial behavior. One study examined gratitude, defined as a positive emotional response in recognition and appreciation of what is meaningful and valuable, often with thankfulness towards something outside oneself^41^. The study measured gratitude, empathy, and prosocial behavior, and found gratitude to be a mediating factor driving empathy induced prosociality^42^. Another study attempted to understand the role of affect in the “identifiable victim effect”. The “identifiable victim effect” refers to the tendency to help identifiable victims rather than anonymous ones. The study found that positive affective valence was associated with promoting the “identifiable victim effect.”^43^.

Researchers exploring the relationship between emotional empathy and prosocial behavior have proposed that positive emotional empathy may be a driver of prosocial behavior. One model of this relationship suggests that perceiving positive affect in others induces vicarious positive affect, which in turn leads to prosocial behaviors. This model asserts that prosocial behavior induces further positive affect^29^.

This model is supported by studies that suggest a positive feedback loop between positive affective states and prosocial behavior. One study examined the association between spending money on others and reported happiness. Participants were asked to recall a memory of spending money on themselves or on others and were subsequently asked to complete happiness measures. When the level of happiness was analyzed, it was found that those who recalled spending money on others displayed higher levels of happiness. Subsequently, participants were given the option to spend money on themselves or others, and it was found that those who were happier were more likely to spend money on others rather than themselves^44^. In another study, participants were asked to engage in a writing activity that was deemed to be positive in nature, with topics including gratitude, positive self-image, or recollection of an intensely positive experience; or, they were asked to write about a neutral topic. Participants were subsequently asked to perform kind acts. The study found that those who participated in positive writing reported increased effort in kind acts they performed; moreover, those that reported greater efforts in kind acts reported greater overall well-being. Another study found that gratitude-induced elevation in affect led to increased effort toward kind acts and that higher effort in kind acts led to further feelings of well-being^45^.

Taken together, the research suggests a complex relationship between empathy and prosocial behavior. It appears that positive affect may not only mediate this relationship, but catalyze a positive feedback loop with prosocial behavior.

## The role of empathy in depression

Empathy may play an important role in the pathophysiology of depression as several studies have found significant association between depressive disorders and deficits in empathy^46–50^. For example, one study found that individuals with major depressive disorder displayed reduced cognitive and emotional empathy in comparison to control subjects^48^. In another study, participants with persistent depressive disorder displayed reduced compassion and social connection with others^47^. A systematic review of thirty-seven studies found empathy to be impaired in depression^50^. Further lending evidence that empathy may play a key role in depression is that therapeutic modalities which improve emotion recognition, empathic concern, and perspective taking such as mindfulness-based interventions, compassion cultivation training (CCT), and attachment-based compassion therapy (ABCT) demonstrate efficacy for the treatment of depression^51–57^.

Contrarily, some research suggests that empathy is positively correlated with depression^58^. This discrepancy may point to the heterogeneity of depression as an illness, with some presentations of depression, such as persistent depressive disorder, treatment-resistant depression, and childhood-onset depression manifesting with relative deficits in empathy^59–61^. It is possible that in some presentations of depression, empathy may not be a significant factor. In other presentations such as depression with concomitant anxiety, empathic response, particularly negative emotional empathy, may worsen distress^62,63^.

## Psilocybin and empathy

Research suggests a link between psilocybin and empathy such that psilocybin may induce changes in specific subtypes of empathy. A double-blind placebo-controlled study showed that healthy volunteers who ingested psilocybin had acute increases in implicit emotional empathy, described as sharing an emotional state with others, and explicit emotional empathy, described as prosocial attitudes toward others, but displayed no changes in measures of cognitive empathy^64^. Importantly, psilocybin may induce increased empathy in the context of depression. For example, in participants with treatment-resistant depression, psilocybin ingestion has been shown to increase emotional facial processing, which is positively correlated with empathy^65–67^. While there is limited research available on the length of effect, some evidence suggests that increased emotional empathy may persist beyond the acute effects of psilocybin. For instance, one study found emotional empathy remained enhanced at seven days, and another suggested increased emotional empathic states at up to 6 months^16,68^.

It is worth noting that other serotonergic psychedelics, which have similar neurobiological and phenomenological mechanisms, have also been shown to enhance empathy. Lysergic acid diethylamide has been shown in at least two studies to enhance empathy and prosocial attitudes^69,70^. Ayuhuasca, a plant that contains the psychedelic compound dimethyltryptamine, has also been shown to increase emotional empathy^71,72^.

## Psilocybin and prosocial behaviors

Some research has examined the association between psilocybin and prosocial behavior. Interest has been largely focused on changes in affective states and reductions in psychiatric symptoms. Behavioral change has not been well studied, with an important exception being substance use, which is an active area of research^4,73^. The current body of research appears to be mixed, with some studies suggesting psilocybin induces prosocial behavior and others showing this may not be the case.

Research in criminal behavior and recidivism suggests there may be an association between psilocybin and prosocial behavior. An observational study found that convicted felons who had a history of psychedelic use were more likely to comply with the rules of community corrective supervision. This sample population included those with a history of substance use and violent offense^74^. A prospective study exploring the relationship between psychedelic use and intimate partner violence in inmates found a lower incidence of intimate partner violence in individuals with a history of hallucinogen use^75^. Although the first study above did not specify which hallucinogens were used, the second found that 86.9% of study participants used classical, serotonergic psychedelics, including psilocybin. These naturalistic study designs can only show associations, but the results suggest that psilocybin and other psychedelics may be protective against antisocial behaviors.

Another interesting area of research explores the association of pro-environmental behaviors and psilocybin. While prosocial behaviors are generally thought to be those that are human to human in nature, prosocial behavior can also be actions benefiting non-human beings such as plants and animals.^77^Pro-environmental behavior can be categorized as a type of prosocial behavior given that it is not self-serving, and rather focuses on contributing positively to beings external to oneself (plants, animals, humans)^76^. In a survey study of 1487 participants, those who endorsed a lifetime history of classic psychedelic use including psilocybin, were more likely to endorse feelings of connectedness to nature and self-reported pro-environmental behaviors^77^. A later follow-up study showed that the potency of the psychedelic experience, specifically mystical experience as measured by the mystical experience questionnaire, was positively associated with self-reported pro-environmental behavior^78^.

Some studies, however, have shown lack of association between psilocybin and prosocial behavior. For example, one study examined both empathy and moral decision making after psilocybin ingestion. It was found that while emotional empathy was enhanced with psilocybin, moral decision making was unchanged^64^. Another study found that psilocybin decreased altruistic punishment, or punishment of others without personal gain, when participants were told their actions would affect another person rather than a computer^79^. While altruistic punishment can be considered prosocial, the reduction was assumed to be due to the social reward of minimizing punishment of another. In that sense, this finding could be interpreted as either inducing or inhibiting prosocial behavior.

These findings suggest that there may be a relationship between psilocybin and prosocial behavior, although this is certainly not definitive. It is possible that there is not a direct relationship between psilocybin and prosocial behavior and that activation of other psychological processes is required in order to observe an association. More robust studies conducted to explore psilocybin and prosocial behavior are necessary to further understand this relationship.

## A proposed mechanism for the enduring effects of psilocybin

**Fig. 1:**
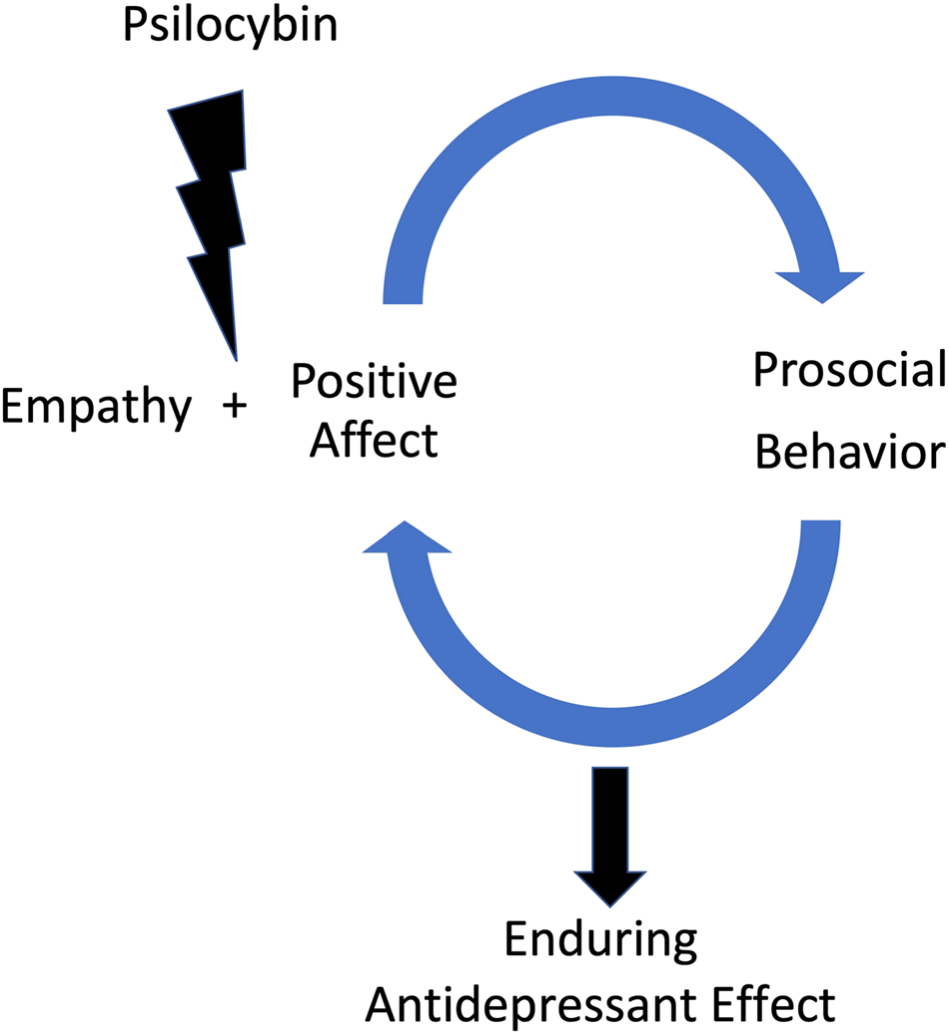
Psilocybin may induce a positive feedback loop involving empathy, prosocial behavior, and positive affect. Psilocybin may lead to enhancement of both empathy and prosocial behaviors in the context of positive affect, inducing a positive feedback loop between positive affect and prosocial behavior that sustains long-term antidepressant effects from a single dose.

In exploring the persisting antidepressant effects of psilocybin, it is intriguing to consider how empathy and prosocial behavior may interact in leading to therapeutic effects. As this paper has summarized, research suggests that psilocybin may lead to increases in empathy and prosocial behavior. Moreover, while not the focus of this article, psilocybin has been shown to produce enduring positive affect^16^. We hypothesize a mechanism involving empathy and prosocial behavior in the context of positive affect which may induce a positive feedback loop, which in part, may lead to the enduring antidepressant effects of psilocybin (see Fig. 1).

This hypothesized model is similar to the one proposed by Telle et al. which was previously discussed^29^. The model proposed by Telle et al. suggests that positive emotional empathy can induce prosocial behavior, which then further leads to positive affect. It is possible that psilocybin induces positive emotional empathy, which may lead to prosocial behavior, followed by a feedback loop involving prosocial behavior and positive affect. This cycle may then be self-propagating, and able to sustain the therapeutic effects of psilocybin after a single dose. It is also possible that psilocybin induces empathy and positive affect distinctly, but simultaneously, which can also produce a similar self-propagating cycle.

It can be argued that positive affect may independently be driving the antidepressant effects of psilocybin, and that empathy and prosocial behavior may not be involved. It can also be argued that positive affect may induce prosocial behavior independent of empathy. While this may be possible, many substances that can produce positive affect, such as illicit amphetamines and alcohol, do not display enduring antidepressant effects, nor do they display positive social effects beyond acute intoxication^80–84^. Moreover, research suggests that even challenging experiences with psilocybin, in which immediate positive affective states are not present, can produce longitudinal positive affect and sustained therapeutic benefit^85^. Thus, while positive affect is likely a component of the therapeutic mechanism, other psychological factors are contributing. As aforementioned, feelings of connectedness with others, which is associated with empathy and prosociality, which have therapeutic value, lend evidence to these processes being involved in the therapeutic mechanism of psilocybin^20,21,24^.

Depression is a heterogeneous illness with certain presentations manifesting with deficits in empathy^59–61^. Hence, this hypothesized mechanism may be a catalyst for psychological change in specific presentations of depression where empathy is impaired. It is also possible that in other variants of depression where impairment of empathy is not present, psilocybin may produce effects through other potential mechanisms or the antidepressant effects of psilocybin may be attenuated.

Psilocybin is currently being studied in conjunction with psychotherapy, termed psilocybin-assisted therapy. However, the modality of psychotherapy used and even the need for a psychotherapy component have been brought into question^86^. Examining the effect of psilocybin on empathy and prosocial behavior could produce vital information for how we can leverage psychological strategies to increase the therapeutic benefits of psilocybin. For example, if psychotherapeutic interventions are framed such as to encourage positive affect and empathy, they may be coupled with psilocybin and work in a synergistic manner. Some research already suggests that this may be the case^87,88^.

Understanding the effect of psilocybin on empathy and prosocial behavior may also be useful in understanding therapeutic effects of psilocybin in other psychiatric disorders. For example, psilocybin has shown promise in the treatment of both obsessive–compulsive disorder and alcohol use disorder and these disorders have been associated with deficits in empathy^4–6,89,90^. In that sense, empathy may be a transdiagnostic component of psilocybin’s therapeutic effect, a possibility that is worth further exploration.

## Conclusion

In this article, we review the literature on empathy and prosocial behavior as it pertains to psilocybin. Currently, research on the effect of psilocybin on empathy and prosocial behavior is limited. However, enough evidence exists to warrant further exploration of this area. Given the current body of evidence, we hypothesize a mechanism by which psilocybin may lead to enduring effects, in part, through a self-propagating feedback loop involving empathy, prosocial behavior, and positive affect. Further research into the effects of psilocybin on empathy and prosocial behavior will help broaden our understanding of psilocybin and other psychedelics as a treatment for psychiatric illness.
